# Genetic Heterogeneity of SLC22 Family of Transporters in Drug Disposition

**DOI:** 10.3390/jpm8020014

**Published:** 2018-04-16

**Authors:** Elisa Lozano, Oscar Briz, Rocio I. R. Macias, Maria A. Serrano, Jose J. G. Marin, Elisa Herraez

**Affiliations:** 1 Experimental Hepatology and Drug Targeting (HEVEFARM), University of Salamanca, IBSAL, 37007 Salamanca, Spain; elisa_biologia@usal.es (E.L.); obriz@usal.es (O.B.); rociorm@usal.es (R.I.R.M.); maserrano@usal.es (M.A.S.); elisah@usal.es (E.H.); 2 Center for the Study of Liver and Gastrointestinal Diseases (CIBERehd), Carlos III National Institute of Health, 28029 Madrid, Spain

**Keywords:** cancer, carrier, chemotherapy, mutation, pharmacology, polymorphism, tumor

## Abstract

An important aspect of modern medicine is its orientation to achieve more personalized pharmacological treatments. In this context, transporters involved in drug disposition have gained well-justified attention. Owing to its broad spectrum of substrate specificity, including endogenous compounds and xenobiotics, and its strategical expression in organs accounting for drug disposition, such as intestine, liver and kidney, the SLC22 family of transporters plays an important role in physiology, pharmacology and toxicology. Among these carriers are plasma membrane transporters for organic cations (OCTs) and anions (OATs) with a marked overlap in substrate specificity. These two major clades of SLC22 proteins share a similar membrane topology but differ in their degree of genetic variability. Members of the OCT subfamily are highly polymorphic, whereas OATs have a lower number of genetic variants. Regarding drug disposition, changes in the activity of these variants affect intestinal absorption and target tissue uptake, but more frequently they modify plasma levels due to enhanced or reduced clearance by the liver and secretion by the kidney. The consequences of these changes in transport-associated function markedly affect the effectiveness and toxicity of the treatment in patients carrying the mutation. In solid tumors, changes in the expression of these transporters and the existence of genetic variants substantially determine the response to anticancer drugs. Moreover, chemoresistance usually evolves in response to pharmacological and radiological treatment. Future personalized medicine will require monitoring these changes in a dynamic way to adapt the treatment to the weaknesses shown by each tumor at each stage in each patient.

## 1. Introduction 

**Figure 1:**
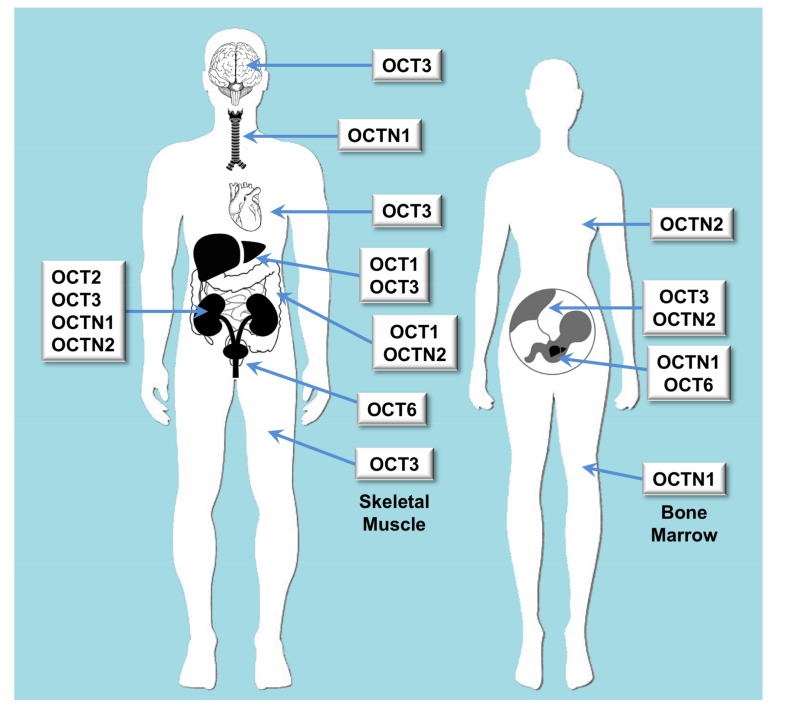
Scheme of the main features regarding organ distribution of proteins belonging to the SLC22 family of transporters able to transport organic cations. OCT: organic cation transporter; OCTN: organic carnitine transporter.

**Figure 2:**
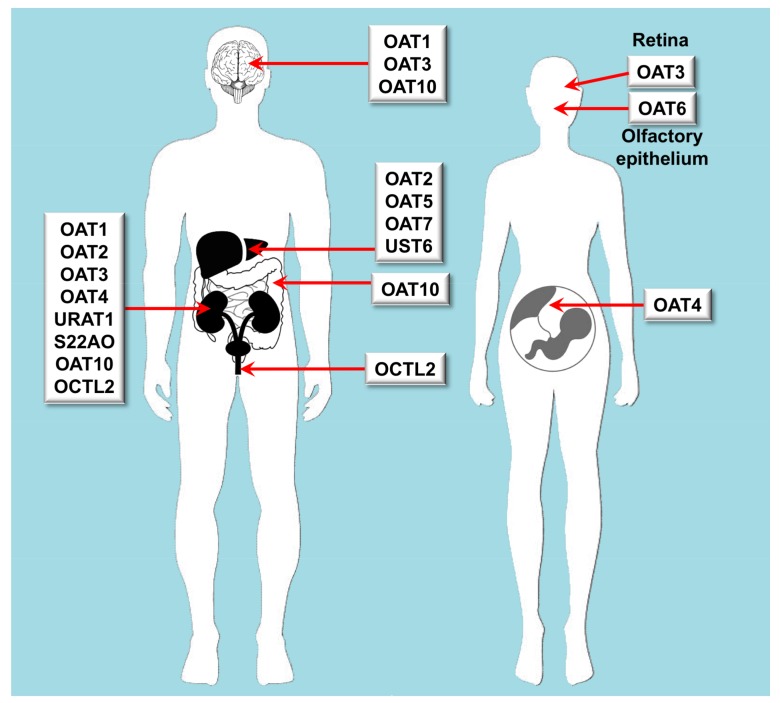
Scheme of the main features regarding organ distribution of proteins belonging to the SLC22 family of transporters able to transport organic anions. OAT: organic anion transporter. OCTL: organic cation transporter-like; S22AO: SLC22A orphan; URAT: urate transporter; UST: unknown substrate transporter.

Owing to its broad spectrum of substrate specificity and strategical expression in organs accounting for drug disposition, such as intestine, liver and kidney, the SLC22 family of human proteins plays an important role in physiology, pharmacology and toxicology (Figure 1 and Figure 2). This family includes 17 well-identified transporters, some orphan transporters and unassigned gene products, still to be fully characterized. Evolutionary analysis indicates that SLC22 transporters fall into at least six subfamilies [1]. In the present review, we will use a traditional and more simplified classification based on the electric nature of the substrates, dividing SLC22 carriers into those able to transport organic cations (OCTs) or anions (OATs). 

All SLC22 proteins share a similar membrane topology [2] consisting of 12 α-helical transmembrane domains (TMD), with three highly conserved areas in their structure that are important for their function: a large extracellular loop with many glycosylation sites between TMD1 and TMD2; a large intracellular loop in the central region between TMD6 and TMD7 with conserved residues for phosphorylation; and some motifs in TMD9 and TMD10 that contain amino acids that are crucial for the transport activity of the protein [3]. No specific residues or domains have been found to determine the specificity of the substrate, suggesting that the three domains work in cooperation with each other [4].

OCT subfamily includes three organic cation transporters (OCT1, OCT2 and OCT3). These are electrogenic facilitative transporters whose function depends on the electrochemical gradient of their substrates (Table 1). The inside-negative plasma membrane potential facilitates the uptake of their substrates, which permits to achieve intracellular concentrations up to 10-fold higher than outside the cells [5]. 

Phylogenetic studies of this gene subfamily suggest that it also contains a clade formed by four plasma membrane transporters of carnitine (OCTN1, OCTN2, OCTN3 and OCT6). These are electroneutral transporters that differ in their affinity for carnitine and other substrates [2] (Table 1). 

The OAT subfamily is also divided into clades [3]. The major clade contains 15 genes, which can be divided into three subclades: the Oat, which is formed by the best functionally characterised organic anion transporters, the Oat-like, and the Oat-related, whose members seem to be able to transport organic cations [3]. The energy driving the function of these proteins is not yet fully understood. Some OATs work as anion exchangers, the driving force for substrate uptake being the outward gradient of intracellular dicarboxylates, generally α-ketoglutarate, but also succinate or even monocarboxylates such as lactate [6]. OATs play their main physiological role in the kidney, although in the liver and other organs some OAT transporters can also participate in the transport of endogenous compounds and xenobiotics (Table 2), playing important roles in pharmacology and toxicology. For instance, there are OAT single nucleotide polymorphisms (SNPs) that affect the sensitivity to several nephrotoxic compounds [7,8]. However, it has been argued that in many cases, SNPs in multiple OATs and apical organic anion transporters may be necessary to bring out a different phenotype [9].

## 2. Carriers for Organic Cations

OCT1 (*SLC22A1*) is mainly expressed at the sinusoidal membrane of hepatocytes, but also in small intestine, lung, placenta, heart, skeletal muscle, brain, eye and immune system cells [10]. Although some weak bases and non-charged compounds can be transported, most OCT1 substrates are organic cations, such as the model substrates tetraethylammonium (TEA), *N*-methylquinine, and 1-methyl-4-phenylpyridinium (MPP), and endogenous compounds, such as catecholamines (dopamine, epinephrine or norepinephrine) [11], choline [2], histamine, serotonin, spermidine [11], putrescine [12], some vitamins, such as thiamine [13] or *N*^1^-methylnicotinamide [14] and the neuromodulator histidyl-proline diketopiperazine [15]; they are rarely organic anions such as prostaglandins E_2_ and F_2α_ [16].

OCT2 (*SLC22A2*) is expressed at the basolateral membrane of tubular epithelial cells of the kidney [17], in several areas of the brain [18]; where it transports biogenic amine neurotransmitters, such as dopamine, and serotonin, and in lung [19]; where it mediates the luminal release of acetylcholine. OCT2 can transport substrates in a bi-directional manner [18,19] and prefers smaller substrates than OCT1. 

OCT3 (*SLC22A3*) has a broad tissue distribution, with high expression in liver, skeletal muscle, heart, kidney and placenta [20,21]; but also in small intestine, brain, uterus, or salivary glands [22]. There is high expression of OCT3 in brown adipose tissue, where it could play a role as regulator of norepinephrine in the extracellular space [23]. Like OCT1 and OCT2, OCT3 transports predominantly organic cations, but also some uncharged molecules.

OCTN1 (*SLC22A4*) is expressed in renal epithelium, bone marrow, trachea and fetal liver and, at a lower level, in other tissues, such as prostate, spleen, spinal cord and eye [24,25]. OCTN1 mediates organic cation transport in a bidirectional and pH-dependent manner. Although it was initially described as a carnitine transporter, the xenobiotic amino acid ergothioneine is now considered its main physiological substrate [26]. OCTN1 has also been involved in acetylcholine transport [27]. Together with the localization at the plasma membrane, OCTN1 has been also detected at the mitochondria [28].

OCTN2 (*SLC22A5*) is predominantly expressed in polarized epithelial cells of intestine, kidney, placenta, and mammary gland, and at lower levels in many other tissues [29]. In the same way as OCTN1, OCTN2 can take up carnitine and other organic cations in a sodium-independent and dependent manner [30]. OCTN2 is considered the main transporter responsible for carnitine homeostasis, participating in its intestinal absorption and tissue distribution.

OCTN3 was first identified in mice. A human orthologue has been identified [31] and localized at the peroxisomes. Accordingly, has been postulated to be a peroxisomal membrane transporter with a unique role in the maintenance of intracellular carnitine homeostasis [32]. OCT6 (*SLC22A16*), also known as CT2, has a unique pattern of tissue distribution, being mainly expressed in testis, hematopoietic cells and fetal liver. OCT6 is able to transport carnitine, but no other typical substrates of OCTs/OCTNs [33].

### 2.1. Role of Organic Cation Transporters in Drug Disposition

Owing to the pivotal role of the liver in the metabolism of endogenous compounds and xenobiotics, impaired function of liver transporters can have an important impact on drug disposition and distribution. The kidney also plays a key role in excreting water-soluble drugs or their derivatives. Inhibition of renal drug transport can affect drug plasma concentrations, leading to altered pharmacological effects and/or adverse drug reactions. Drug–drug interactions due to cross-inhibition of plasma membrane transporters, leading to impaired elimination/uptake of one drug in the presence of another, are frequently responsible for altered pharmacokinetic and problems of safety and efficacy. The International Transporter Consortium included OCTs/OCTNs among the transporters clinically important in drug absorption and disposition, and recommended in vitro methods to study transporter–drug interactions [34].

Among many drugs that can be transported by OCT1 the most relevant ones are shown in Table 1 and commented below. Metformin used for the treatment of type II diabetes, is taken up by the liver and intestine via OCT1. Metformin was the first described OCT1 substrate. [35]. The anti-retroviral lamivudine, used to prevent human immunodeficiency virus (HIV) replication, is efficiently taken up by OCT1 [36]. Because this transporter is also expressed in mononuclear cells of the lymph nodes, it has been proposed that it could participate in the accumulation of lamivudine in lymph nodes of HIV-infected patients. Zalcitabine, another anti-retroviral drug, is also taken up by OCT1 [36]. *O*-desmethyltramadol is an opioid analgesic and the main active metabolite of tramadol. OCT1 is involved in the uptake of this drug by the liver, which is a key factor affecting the efficacy of tramadol treatment [37]. OCT1 is a high-capacity transporter of the anti-migraine drug sumatriptan [38] and the anti-emetics tropisetron and ondansetron. Impaired OCT1 function may increase the efficacy of these drugs by limiting their hepatic clearance [39]. Assays carried out both in vitro and *in vivo* suggest that OCT1 mediates morphine uptake by hepatocytes and that the lack of OCT1 activity results in significantly higher concentrations of morphine in plasma. In contrast to morphine, codeine is an inhibitor, but not a substrate of OCT1 [40]. Experiments using *Xenopus laevis* oocytes demonstrated that OCT1 transports histamine-2 (H2) receptor antagonists. Thus, OCT1 is likely to play a major role in the intestinal absorption and hepatic disposition of the H2 receptor antagonists ranitidine and famotidine [41]. Moreover, in vitro studies have demonstrated that the H2 receptor antagonist cimetidine [42], the anti-arrhythmic quinidine [43], antibiotics of the fluoroquinolone family, such as ciprofloxacin [44] and the anti-convulsant drug lamotrigine [45] are also substrates of OCT1.

OCT2 is also involved in metformin disposition. This drug is mainly eliminated unchanged in urine after tubular secretion, which is mediated by OCT2 [46]. Sulpiride is a selective dopamine D2 receptor blocker used in the treatment of patients with schizophrenia and depression. OCT2 also accounts for the uptake of this drug by proximal tubular cells from the bloodstream [47]. The anti-malarial drugs proguanil and cycloguanil, the anti-retroviral entecavir, and anti-hypertensive atenolol are also substrates of OCT2, while amodiaquine, pyrimethamine and quinine are inhibitors of this transporter [48,49,50]. Cimetidine, anti-arrhythmic drugs quinidine and procainamide, and the anti-retroviral amantadine, are also inhibitors of both OCT2 and OCT1 [51]. Moreover, OCT2 and/or OCT3 mediate the accumulation of fluoroquinolones in renal proximal tubule cells [52].

Due to the ubiquitous expression of OCT3, it has been suggested that this transporter can play a role in drug distribution and elimination. In fact, data obtained in Oct3 knockout mice demonstrated that this transporter also participates in the absorption and elimination of metformin, and determines its bioavailability, clearance, and pharmacologic action [53]. The high expression of OCT3 in salivary glands is responsible for metformin accumulation and secretion into saliva, which could explain its known effect in taste disturbance [22]. *Scutellaria baicalensis* is a popular plant, used in Chinese traditional medicine for the treatment of inflammation, hypertension, and infections. One of its components, wogonin, impairs cellular influx of drugs into renal tubular cells via OCT3 inhibition [54]. Because OCT3 is expressed at the apical membrane of enterocytes, this carrier can also affect the bioavailability of drugs by modifying their intestinal absorption. Although the cationic drug salbutamol, a beta-2 agonist used as anti-asthmatic, has been reported to be a substrate of OCTs, and OCT3 is highly expressed at the airway epithelium, there is evidence suggesting that salbutamol enters bronchial smooth muscle cells via a transporter-independent way. Surprisingly, salbutamol uptake is affected, through an unknown mechanism, by corticosterone and beclomethasone [55].

OCTN1 and OCTN2 participate in both renal secretion and reabsorption of sulpiride [47] and entecavir [49], and could mediate the efflux of fluoroquinolones into urine after being accumulated in renal proximal tubule cells [52]. OCTN1 also mediates the renal secretion of gabapentin, a drug used to treat seizures, and post-herpetic neuralgia [56].

Regarding OCT6, little is known about its role in drug disposition. Only interaction with anti-tumor drugs has been described. Thus, decreased OCT6 expression in lung cancer cells has been associated with a reduced intracellular uptake of cisplatin and oxaliplatin, and concomitant enhanced resistance to these platinum-containing drugs [57].

### 2.2. Impact of Genetic Variability in Pharmacokinetics

The gene encoding OCT1, i.e., *SLC22A1*, is highly polymorphic. Approximately 10% of Caucasians are homozygous carriers and 40% are heterozygous carriers of a loss-of-function OCT1 allele [39]. Two of the OCT1 polymorphic variants (OCT1-C88R and OCT1-G465R) have been observed only in combination with the variant OCT1-M420del. The haplotype combining OCT1-C88R and OCT1-M420del is designed allele OCT1*6, whereas the haplotype with OCT1-G465R and OCT1-M420del is designed allele OCT1*5. Moreover, shorter OCT1 isoforms originated by aberrant splicing due to exon skipping and intron retention sequences are also expressed. These OCT1 variants result in truncated nonfunctional proteins [58].

Several OCT1 variants have been associated with impaired/reduced drug uptake (Table 3). Thus, genetic variation in OCT1 has been associated with the pharmacokinetic performance of metformin. On one hand, a clinical study in healthy volunteers indicated that plasma glucose levels after metformin treatment were higher in people carrying OCT1 polymorphisms (OCT1-R61C, G401S, M420del, and G465R which exhibited reduced uptake of metformin in the cellular assays) than in those carrying the wild-type OCT1 sequence. This may be related to dynamic differences in metformin levels in the intestine between individuals with and without these variants, which may affect metformin absorption [59]. In a clinical study with type-II diabetes mellitus patients only the intronic variant *rs622342* of all studied variants was associated with the glucose-lowering effect of metformin [60]. In addition to direct efficacy of metformin, OCT1 variants have been associated with the secondary effects of this drug. Thus, the variant c.1276 + 1insGTAAGTTG (*rs36056065*), which consists of an 8-bp insertion of intron 7 between exons 7 and 8, results in a truncated protein that has been associated with adverse gastrointestinal side effects in patients treated with metformin [61]. It has been hypothesized that metformin intolerance in these patients is induced by a local increase of drug concentrations in the intestine. The association of OCT1 variants and gastrointestinal side effects of metformin therapy was also identified in patients with OCT1-M408V variant [61], although it had been described that this SNP mediates normal metformin uptake *in vitro* [59]. Disposition of morphine could be also modulated by OCT1 genotypes. Thus, in vitro studies indicated that OCT1 variants OCT1-G401S, OCT1*5 and OCT1*6 were not able to transport morphine, and M420del and R61C variants showed very limited transport capacity compared to wild-type OCT1. When the effect of these SNPs was studied in clinical assays, it was found that the presence of these polymorphisms resulted in higher morphine plasma concentrations due to lower hepatic clearance [40]. Another study indicated that OCT1 genotypes largely determine the pharmacokinetics of intravenously injected morphine. Defective OCT1 variants potentially lead to a reduced clearance of morphine and consequently to a higher frequency of drug-induced toxicity episodes [62,63].

The anti-emetic drug tropisetron is primarily metabolized in hepatocytes after been taken up through OCT1. Variants of this transporter have been postulated to alter tropisetron response. SNPs that reduce OCT1 function, such as R61C, G401S, M420del, OCT1*5 and OCT1*6, have been associated with increased tropisetron plasma concentrations due to poorer hepatic clearance and consequently with a better response to this treatment in patients bearing these variants [39]. Reduced OCT1 function may be expected to increase the efficacy of ondansetron, another antiemetic drug, by limiting their hepatic clearance; however, clinical assays did not show a significant association between impaired OCT1 function and a better response to this drug [39].

In a population-based cohort study, the polymorphism c.1386A>C (*rs622342*), localized in a non-coding region of the *SLC22A1* gene, was associated with higher prescribed doses of anti-Parkinson drugs and a shorter survival after start of levodopa therapy [64]. There are two possible explanations for this association: (i) it is possible that the *rs622342 C* allele accounts for a reduced anti-Parkinson drug uptake by the small intestine and hence is responsible for a lower bioavailability [65]; (ii) OCT1, which is expresses at low levels in the brain, can act as rate-limiting step for drug uptake by this target tissue. 

In a cohort of Chinese patients with epilepsy, the OCT1-M408V (c.1222A>G) polymorphism was significantly associated with serum concentrations of lamotrigine. Results indicated that these were 26% lower in patients with GG genotype compared with AA and AG genotypes. These findings suggested that patients with GG genotype required higher maintenance dose of lamotrigine to achieve similar plasma levels [66]. These effects could be due to OCT1-mediated hepatic uptake of this drug and/or to the fact that, as has been demonstrated in in vitro assays, lamotrigine is taken up by brain endothelial cells through OCT1 [66].

Results from in vitro experiments have revealed that some OCT1 polymorphisms (R61C, G401S, M420del, OCT1*5 and OCT1*6) result in impaired *O*-desmethyltramadol transport by this carrier. Clinical data indicate that healthy volunteers carrying these variants with loss of function, also present higher plasma concentrations of the *O*-desmethyltramadol together with high concentrations in the central nervous system, which is probably due to reduced hepatic clearance of this drug [37].

OCT1 may be involved in the transport of the antidepressants amisulpride and sulpiride in the brain. In vitro studies demonstrate a strong reduction of the OCT1-mediated uptake of both drugs by the five most common OCT1 loss-of-function alleles (R61C, G401S, M420del, OCT1*5 and OCT1*6 [67].

OCT1-mediated uptake is a limiting step in the hepatic metabolism of sumatriptan. Clinical data indicated that genetically determined loss of OCT1 activity (OCT1-R61C, C88R, G401S, G465R) results in two-fold increased systemic exposure to sumatriptan [38].

OCT1-C88R and G465R expressing cells present complete loss of lamivudine uptake in vitro [68]. Also in vitro assays have revealed that polymorphic OCT1 variants alter the uptake of the antihypertensive drug debrisoquine. Thus, the SNPs OCT1-C88R and G465R result in complete lack of debrisoquine OCT1-mediated uptake [69].

Described OCT2 SNPs (Table 3) have not been associated to changes in expression, but some induce reduced activity. Lower renal clearance of metformin has been found in patients with OCT2 variants T199I, T201M, and A270S, which lead to higher peak in plasma concentration after metformin administration [70].

The splice variant OCT2-A, although maintains 81% amino acid identity with the wild-type protein, displays different affinity for several organic cations. Because OCT2-A is mainly expressed in kidney, it is believed that the expression of OCT2-A affects the renal clearance of its substrates [71].

Among five genetic variants of OCT3 functionally characterized [72], A116S, T400I, and A439V (Table 3) have a reduced transport activity. A higher susceptibility of hypertension, allergic diseases and psychiatric disorders in subjects bearing these genetic variants has been reported and suggested to be associated with a reduced local clearance of histamines and neurotransmitters in the nervous system.

At least 15 SNPs of OCNT1 have been characterized (Table 3). A reduced transport activity was reported for G462E [73] and M205I [74], while variants D165G and R282X resulted in complete loss of transport function [74]. In Chinese and Indian populations, eight SNPs have been described [75]. Four of these variants: R63H, R83P, G482D, and I500N presented a reduced uptake capacity. Moreover, a reduced tubular secretion of gabapentin in patients with the L503F variant was found [56].

OCNT2 is highly polymorphic (Table 3). Altered transport activity of this carrier has been found in 8 out of 20 genetic OCNT2 variants characterized. Mutations M352R and P478L were associated with loss of transport function despite the protein expression is not impaired in individuals bearing these mutations [76].

## 3. Carriers for Organic Anions 

Some members of the SLC22 family, globally designed here with the classical denomination of OATs, can transport a broad variety of anionic endogenous metabolites and xenobiotic molecules, including many drugs and consequently, they have an important impact on pharmacokinetics [77,78] (Table 2). Most OATs are highly expressed in human kidney and/or liver, and at lower levels in brain, placenta, prostate and testis [77]. OAT1 (*SLC22A6*), OAT2 (*SLC22A7*) and OAT3 (*SLC22A8*) are located at the basolateral membrane of the renal proximal tubular cells, where they are involved in the secretion of drugs and toxins for subsequent elimination into urine [79]. In contrast, OAT4 (*SLC22A11*), OAT10 (*SLC22A13*) and urate transporter 1 (URAT1) (*SLC22A12*) are expressed at the apical membrane of the proximal tubular cells, and are involved in the reabsorption of substances from the tubular fluid [79]. OAT2, OAT5 (*SLC22A10*) and OAT7 (*SLC22A9*) are located at the sinusoidal membrane of hepatocytes and are involved in liver detoxification processes [80]. Other OATs have less pharmacological relevance, such as OAT6 (*SLC22A20*), which is mainly expressed in the olfactory mucosa, but not in the kidney and liver [81], and the SLC22A orphan (S22AO) (*SLC22A24*), the unkwown substrate transporter 6 (UST6) (*SLC22A25*) and organic cation transporter-like 2 (OCTL2) (*SLC22A14*) that are poorly known.

### 3.1. Role of Organic Anion Transporters in Drug Disposition

OAT1 plays an essential role in the renal elimination of a broad range of toxins, drugs such as angiotensin-converting-enzyme inhibitors such as captopril [82], angiotensin II receptor blockers, such as olmesartan [83], diuretics such as furosemide [84], numerous guanine-based antiviral drugs including acyclovir, adefovir, cidofovir, ganciclovir or tenofovir [85,86,87,88], cimetidine and ranitidine [89], and anti-cancer agents, such as methotrexate [90]. In addition, some drugs, such as probenecid [91,92] and nonsteroidal anti-inflammatory drugs (NSAIDs) [91,93,94] bind to OAT1, but cannot be translocated, thus blocking the secretion of another substrates of this transporter. Inhibition of OAT1-mediated renal anion secretion could be used to induce beneficial drug–drug interactions to either enhance activity of antibiotics or reduce renal accumulation and nephrotoxicity of certain antiviral drugs [95,96]. Thus, the co-administration of probenecid with cidofovir has been suggested to protect patients against cidofovir-induced nephrotoxicity, which is associated to excessive drug accumulation in renal proximal tubule cells [95].

OAT2 transport a variety of endogenous organic anions including prostaglandin E_2_, α-ketoglutarate and cAMP [97,98]. Owing to its expression in both liver and kidney, and its ability to transport and hence affect the disposition of a wide variety of pharmacologically active agents, OAT2 is an important determinant in drug disposition. Thus, several antineoplastic drugs interfere with OAT2-mediated transport, whereas others such as methotrexate [97] or irinotecan [99] are substrates of this transporter. Controversial data regarding OAT2-mediated 5-fluorouracil (5-FU) transport have been reported [99,100]. In metastatic colorectal cancer, high expression of OAT2 seems to be a predictor of effectiveness of 5-FU-based chemotherapeutic regimens such as FOLFOX (5-FU/leucovorin/oxaliplatin) [101]. Acyclovir, ganciclovir and penciclovir are also substrates of OAT2 [86]. This also mediates the hepatic uptake of entecavir, an essential nucleoside analogue used in chronic hepatitis B treatment, playing an important role in the mechanisms underlying the efficacy of antiviral treatment. OAT2 is also involved in the transport of cimetidine and ranitidine [102] and some antibiotics, such as erythromycin [103] and tetracycline [104]. In general, NSAIDs are inhibitors of OAT2, but are not translocated [105], except for diclofenac, which has been reported to behave as a specific OAT2 substrate [106]. In addition, several diuretics inhibit OAT2 [84], but whether they are also translocated by this carrier is unknown.

OAT3 is another important mechanism for urinary excretion of circulating anionic drugs and their metabolites. The substrate specificities of both transporters are partly overlapped, but not identical. In general, substrates of OAT3 are bulkier and more lipophilic than those of OAT1. OAT3 can transport zidovudine [107], cimetidine [102], diuretics, such as bumetanide or furosemide [84], antibiotics, including several cephalosporins [82], ciprofloxacin [108,109] and benzylpenicillin [110], statins, such as rosuvastatin [111] and pravastatin [112], and methotrexate [113]. OAT3 also shares with OAT1 many inhibitors, such as NSAIDs [114,115,116], ciprofloxacin [117], probenecid [116] or the integrase inhibitor cabotegravir [118]. Although this protein does not translocate them, they are frequently the cause of drug–drug interactions. Studies on Oat1 and Oat3 knockout animals have revealed a whole new range of endogenous substrates, which suggests that there is less overlap between Oat1 and Oat3 specificity (at least for metabolites) than previously thought [119,120].

Estrone-sulfate is the prototypical substrate of OAT4, but this carrier can also transport other sulfate steroids such as dehydroepiandrosterone sulfate and the purine metabolite urate [121]. Owing to its location, OAT4 can either take up drugs from the tubular fluid or secrete them into urine. Well characterized OAT4 substrates include zidovudine [88], the antibiotic tetracycline [88], methotrexate [113], the NSAIDs ketoprofen and salicylate [93] and the diuretic bumetanide [84]. Other diuretics, such as hydrochlorothiazide and torsemide have been reported to *trans*-stimulate OAT4-mediated transport, suggesting that they can also be translocated [122]. Thus, OAT4-mediated efflux of some diuretic drugs increases urate reabsorption by the kidney leading to hyperuricemia [122].

There is little information available on the physiological role and the pharmacological impact of OAT5. A few drugs have been tested for potential OAT5-mediated transport and all of them have been found to act as inhibitors of this carrier [123].

OAT6 is a multi-specific organic anion transporter preferentially expressed in nasal epithelial cells [81] able to interact with a variety of small-molecule organic anions of physiological significance, among them the odorant molecules constitute the best ligands of this transporter [124]. Regarding its ability to transport drugs, OAT6 has been reported to mediate the uptake of the tyrosine kinase inhibitor sorafenib in human epidermal keratinocytes [125]. This suggests that OAT6 could be involved in the skin toxicity of this anticancer agent. Other therapeutic compounds, including antibiotics, antiviral agents and NSAIDs have been tested on murine Oat6, although none of them has turned out to be substrate of this transporter.

OAT7 transport sulfate steroids such as estrone-sulfate and dehydroepiandrosterone sulfate across the sinusoidal membrane of hepatocytes [126]. OAT7 may play a role in the hepatic uptake of pravastatin [127], the only drug identified as potential substrate of OAT7.

OAT10, previously known as ORCTL3 (organic cation transporter like 3), is an exchanger that takes up extracellular urate or nicotinate coupled to intracellular lactate, succinate, or glutathione [128]. Several diuretics have been reported to act as OAT10 inhibitors, without being themselves transported by OAT10. The immune suppressant cyclosporine A, in addition to inhibiting OAT10, *trans*-stimulate its function, suggesting that this compound is actually transported by OAT10 [128].

URAT1 is responsible for the reabsorption of uric acid from the kidney proximal tubules, thereby playing a key role in uric acid homeostasis [129]. URAT1 has limited substrate specificity, and no drugs transported by URAT1 have been identified. However, URAT1 has been proposed as a target of uricosuric drugs, such as 6-hydroxybenzbromarone, probenecid, indomethacin and salicylate, used to inhibit urate reabsorption [130]. Owing to nephrotoxicity of many anticancer regimens, urate handling is certainly important in oncologic patients undergoing chemotherapy. In this respect, SNPs in several OAT isoforms involved in urate transport [131,132] may favor the appearance of hyperuricemia during treatment.

### 3.2. Impact of Genetic Variability in Pharmacokinetics

Owing to the importance in drug disposition of OATs, the presence of genetic variants has often pharmacological consequences [76,133] (Table 4). The available data regarding the functional relevance of mutations have permitted to distinguish three regions of interest: (i) the large extracellular loop; (ii) the central intracellular loop, highlighting its signature ‘ESPXR’, whose mutations can alter the interaction of the transporter with its substrates; and (iii) the transmembrane domains, especially TMD9 and TMD10, whose mutations may affect the interaction with plasma membrane components [3].

OAT1 and OAT2 have low genetic variability, which may be due to strong negative selection related to the important physiological role played by these carriers [134,135]. Polymorphisms found in the coding region of both OAT1 [134] and OAT2 [135] do not remarkably contribute to inter-individual variability in drug disposition. Although some of the mutations described in these transporters result in loss of activity when expressed in an in vitro system, no effect of these genetic variants in in vivo drug disposition has been found. This is probably due to the overlapping substrate specificity among SLC22 carriers. Thus, OAT1-R454Q variant shows a complete loss of activity when expressed in *X. laevis* oocytes, but no altered renal clearance of adefovir in patients carrying this genetic variant has been found [134]. Similarly, although the variant OAT1-R50H can transport the antivirals adefovir, cidofovir and tenofovir with higher affinity than the wild-type form [87], and therefore it is expected to increase the uptake by epithelial cells of proximal tubules, nephrotoxicity induced by these antiviral drugs is not higher in patients carrying this SNP [134].

An OAT2 splice variant containing an additional nucleotide sequence of the intron 1 (TCCCAG) between exons 1 and 2 of the OAT2 ORF has been detected in healthy liver, kidney and pancreas at approximately the same proportion of expression as the wild-type transporter [136]. The peptide encoded by the spliced variant contains two additional amino acids, Ser and Gln, and is not inserted at the plasma membrane, remaining intracellularly and consequently lacks transport activity [136]. Changes in the proportion of expression between the inactive variant and the wild-type form under physiological and pathological circumstances and its impact in pharmacology have not been elucidated yet.

At least 10 missense mutations in *SLC22A8* gene have been found [137]. Among them, OAT3-R277W and OAT3-I305F variants that affect the central intracellular loop of the protein result in reduced ability to transport OAT3 substrates, such as estrone-3-sulfate and cimetidine [137]. OAT3 activity was completely abolished in OAT3-I260R variant [137]. Clinical studies of the repercussions of these mutations have only been carried out with patients bearing OAT3-I305F variant, who have a significantly lower renal clearance of cefotaxime compared to subjects who were homozygous for the reference allele [138]. Two other inactivating mutations affecting TMD3 (OAT3-R149S) and TMD6 (OAT3-N239X) have been described [137]. Although there is no clinical data available yet, these variants are probably associated with reduced renal elimination of drugs transported by OAT3 [139].

Among non-synonymous SNPs described in the *SLC22A11* gene, OAT4-L29P, OAT4-R48Y, OAT4-V155G (which affect the large extracellular loop) and OAT4-T392I variants have a reduced transport activity [140]. A clinical study has shown that OAT4 genetic polymorphisms with impaired activity are associated to altered renal clearance of the diuretic torsemide [141].

Regarding *SLC22A9* gene, three rare missense mutations have been found (c.268C>T, c.1298C>G/T, and c.1437A>G), which do not affect the hepatic expression of OAT7, but decrease its activity when expressed in vitro, and may alter the in vivo disposition of pravastatin [127].

URAT1 is the only transporter of the SLC22 family whose mutations have been directly related to a disease. Inactivating mutations of URAT1 inhibit the renal reabsorption of urate and cause familial idiopathic hypouricemia [129]. Nonsense (c.774G>A and c.889C>T) and frameshift mutations (IVS2 + 1G>A and c.1639_1643delGTCCT) in the *SLC22A12* gene, which abolish the transport activity, and missense mutations (c.269G>A, c.412G>A, c.490G>A, c.650C>T, c.1145A>T, and c.1289T>C), which reduce its activity, have been described [142]. These variants may decrease renal secretion of drugs transported by URAT1 and, therefore, increase its clearance. However, to date there is no affected drug identified [130].

Usually, the presence of polymorphisms in non-coding regions of the SLC22 genes is not an important factor affecting the expression of these proteins and their transport activity [143,144]. However, a mutation found in the 3′-UTR region of *SLC22A7* gene (c.1592 + 206A>G) results in OAT2 up-regulation and hence, increased uptake of drugs, such as anthracyclines and 5-FU. Consequently, the presence of this mutation has been associated with anthracycline-induced cardiotoxicity [145] and severe toxicity to the 5-FU prodrug capecitabine [146].

## 4. SLC22 Genetic Heterogeneity in Cancer Pharmacology

The set of transformations suffered by cells during oncogenic phase is accompanied by modifications in cell metabolism that also affect the expression of drug transporters, which may have an important impact on the effectiveness of chemotherapy [147]. The expression of members of the SLC22 family in different types of tumors has been well characterized [77]. Pharmacogenomic studies have shown that polymorphisms in genes related to drug transport through the SLC22 family have a considerable effect on the response to different antitumor drugs [148]. Moreover, the allelic variants found in members of the SLC22 family of transporters are associated with various modifications that determine aspects of the disease, such as tumor progression [149].

The mutations described in the *SLC22A1* gene affect all regions of this gene. Restricting the analysis to the coding sequence, one deletion of three base pairs (M420del), eight nonsense mutations, and 49 missense mutations have been reported [58,150,151]. Under physiological circumstances, variants with reduced transport function have substitutions in amino acids that cause more drastic structural changes, being evolutionarily less favorable than the variants with conserved function [152]. However, the situation is the opposite in cancer cells exposed to chemotherapeutic challenge. Because several antitumor drugs, such as irinotecan, mitoxantrone, oxaliplatin, paclitaxel, imatinib and sorafenib have been described as OCT1 substrates [153], the expression level and the activity of this transporter may play a key role for chemotherapeutic response to these drugs. In these sense, in patients with chronic myeloid leukemia (CML), homozygosis for the variants *rs4646278* (R287G/W, c.859C>G) and *rs4646277* (P283L, c.848C>T) have been described together with three low frequency allelic variants: *rs12208357* (R61C, c.181C>T), *rs72552763* (M420del, c.1260_1262delGAT) and *rs683369* (L160F, c.480G>C). CML patients carrying the OCT1 c.480G allelic variant have lower clearance of imatinib than patients homozygous for the c.480C allelic variant [154]. However, clinical data indicates that CML patients with OCT1 polymorphisms that were negatively related with imatinib efficacy had no impact on nilotinib efficacy or toxicity [155]. 

Two OCT1 variants (A99T and G174S) have been identified in patients with chronic lymphocytic leukemia (CLL). Genetic heterogeneity appears for the most common variants of *SLC22A1* in patients with CLL except for the small increase in the allelic frequencies of relatively rare variants such as G38D and P341L [156].

In liver tumors, the expression levels of OCT1 may be important for the response of these patients to sorafenib, the only drug moderately useful in the treatment of hepatocellular carcinoma. So, the expression levels of OCT1 have been described to be downregulated in liver cancer, and also several, variants of OCT1 have been identified in these tumors, including SNPs and splicing variants [151]. Among these SNPs, some of them have been reported in in vitro studies to attenuate sorafenib-induced toxicity; as an example of OCT1 SNPs related with lower sorafenib transport are OCT1 R61S fs*10 and C88A fs*16 that produce a shift in the open reading frame that result in non-functional truncated proteins. In detail, R61S fs*10 is a genetic variant caused by a frameshift mutation originated by deletion of two nucleotides and the insertion of one (c.181delCGinsT), that generates a change in the amino acid 61 (arginine changes to serine) and also introduces a stop codon that makes that the protein ends 10 amino acids after of position 61, making a resulting protein of 71 instead of 554 amino acid that form the wild type OCT1. Similarly, C88A fs*16 is caused by a deletion of one nucleotide (c.262delT), that generates a change in the amino acid at the position 88, and also introduces a stop codon that makes that the resultant protein contain only 104 amino acids. Short and non-functional OCT1 variants have also been detected in glioma [58]. 

Loss of expression of OCT2 at the transcriptional and protein level in renal carcinoma cells has been reported [147]. In vitro studies have indicated that the repression of OCT2 is an important factor in the resistance to oxaliplatin in renal carcinoma cells, and a non-synonymous SNP (*rs316019*) in the *SLC22A2* gene has been associated with a decrease in cisplatin nephrotoxicity in patients with renal carcinoma [157]. SNPs in OCT2 in combination with ENT1 and MATE1 variants may serve as predictive and prognostic markers in refractory metastasis of colorectal cancer treated with TAS-102 [158]. 

OCT3 SNPs have been related to the level of *SLC22A3* mRNA and the risk of prostate, colorectal cancer and other diseases [159]. Thus, in colorectal cancer, an OCT3 variant located at 6q26-q27 (*rs7758229*; c.975 + 8374G>A in the non-coding region of *SLC22A3*), has been associated with distal tumors [160]. The polymorphic variant *rs9364554* (c.582 + 1786C>T) is associated with decreased OCT3 expression in prostate cancer [161], whereas the common variant g.-2G>A (*rs555754*) has been associated with a higher level of OCT3 expression [159]. 

A relationship between variants of OCTN transporters and the efficacy of imatinib has been suggested to play an important role in the efficacy of this drug in adjuvant therapy [149]. Among patients with gastrointestinal stromal tumors treated with imatinib, the time to progression period significantly improves in carriers of the C allele of the variant of OCTN1 *rs1050152* (L503F, c.1507C>T) as well as in carriers of the minor alleles of the variants of OCTN2 *rs2631367* (c.-207C>G) and *rs2631372* (c.-2087G>C), both localized in the promoter, suggesting that the activities of OCTN1 and OCTN2 may be predictors of efficacy to chemotherapy with imatinib [147].

Pharmacogenetic studies have shown that polymorphisms in genes related to metabolism and drug transport can have a significant effect on the response to imatinib in patients with CML. Thus, an important association between the polymorphism *rs1050152* of OCTN2 and the molecular response to imatinib has been found [162]. Variants *rs2631367* (L503F, c.1507C>T) and *rs2631372* (c.-2087G>C) in OCTN2 are associated with an improvement in time to progression in CML [162].

There is a surprising high promiscuity in the profile of endogenous and xenobiotic compounds transported by the different OATs, so it is critical to understand the mechanisms that regulate the expression and function of OATs by genetic and epigenetic factors [163]. In addition, inter-individual variability among patients and tumors, which can markedly affect the outcome of chemotherapy, is largely dependent upon SNPs occurrence [164,165]. Thus, OAT2, which is highly express in liver, play a role in the uptake and subsequent metabolism of many antineoplastic drugs, such as 5-FU, which is used in colorectal and pancreatic cancer therapy. 

In addition to SNPs, epigenetic mechanisms have been related with changes in the expression levels of SLC22 genes in different tumors. Thus, hypermethylation has been proposed as an important event related with a decrease in the expression of several SLC22 genes. For instance, OCT1 down-regulation in hepatocellular carcinoma has been related with an enhanced methylation of the promoter [166]. Similarly, methylation is also involved in the inhibition of OCT2 transcription. Consistently, epigenetic activation of OCT2 by decitabine, a demethylating agent, has been observed in renal carcinoma cells sensitive to oxaliplatin in a sequential and combinatorial therapy carried out in vitro and in a xenograft model [157]. Hypermethylation of the promoter region of *SLC22A3* gene is an important mechanism accounting for reduced OCT3 expression in prostate cancer [159].

## 5. Conclusions and Perspectives

In the time of emerging personalized medicine, it is becoming more evident that there is a need of, not only understanding the mechanisms of action and the profile of substrate specificity characteristic of each transporter involved in drug disposition, but also identifying the genetic signature accounted for by the existence of polymorphisms that markedly determine the response of the patient to any given drug or combination of drugs. In this respect, the SLC22 family of transporters constitutes an important subject of interest for several reasons that have been reviewed here. In the near future, modern pharmacology will probably need to implement an analysis of expression levels and the detection of genetic variants regarding SLC22 transporters and other carriers playing a key role in drug disposition, such as members of the SLCO family of uptake transporters and ABC export pumps. One could expect this analysis to be performed before treatments, but in some cases this should be repeated during certain treatments, which will be most necessary in anticancer chemotherapy, due to the dynamic nature of the target tissue, in which both expression levels and mutation may undergo alterations as an individual cancer characteristic present before starting the treatment but that usually change during chemotherapy or even radiotherapy of the tumor. 

## Figures and Tables

**Table 1:** Characteristics of the human organic cation transporters of the SLC22A family.

Major Clade	Subclade	Gene	Protein	Tissue Distribution	Endogenous Substrates	Xenobiotic Substrates/Inhibitors
OCT	Oct	*SLC22A1*	OCT1	Liver and to a less extent also in brain, heart, immune cells, intestine, kidney and lung	Biogenic monoamines, biogenic polyamines, catecholamines, ethanolamines, neuromodulators, vitamins, prostaglandins	Cimetidine, ciprofloxacin, famotidine, lamivudine, lamotrigine, metformin, O-desmethyltramadol, ondansetron, quinidine, ranitidine, sumatriptan, tropisetron, zalcitabine
OCT	Oct	*SLC22A2*	OCT2	Kidney, brain, lung	Acetylcholine, dopamine, serotonin	Amantadine, anti-malarials, atenolol, cimetidine, entecavir, fluoroquinolones, metformin, procainamide, quinidine, sulpiride
OCT	Oct	*SLC22A3*	OCT3	Widely expressed	Norepinephrine	Metformin, wogonin
OCT	Octn	*SLC22A4*	OCTN1	Kidney, bone marrow, trachea, fetal liver (at lower levels in many tissues)	Acetylcholine, carnitine	Ergothioneine, entecavir, fluoroquinolones, gabapentin, sulpiride
OCT	Octn	*SLC22A5*	OCTN2	Intestine, kidney, placenta, mammary gland	Carnitine	Entecavir, fluoroquinolones, sulpiride

**Table 2:** Characteristics of the human organic anion transporters of the SLC22A family.

Major Clade	Subclade	Gene	Protein	Tissue Distribution	Endogenous Substrates	Xenobiotic Substrates/Inhibitors
OAT	Oat	*SLC22A6*	OAT1	Kidney, brain	cGMP, C5- and C6-mono and dicarboxylates, prostaglandins, urate	Acyclovir, adefovir, captopril, cidofovir, cimetidine, furosemide, ganciclovir, methotrexate, olmesartan, tenofovir, ranitidine
OAT	Oat	*SLC22A7*	OAT2	Liver, kidney	Conjugated steroid hormones, cGMP, nucleobases, nucleosides, nucleotides, prostaglandins	Acyclovir, cimetidine, diclofenac, entecavir, erythromycin, ganciclovir, irinotecan, methotrexate, penciclovir, ranitidine, tetracycline, 5-FU
OAT	Oat	*SLC22A8*	OAT3	Kidney, retina, brain	Acidic neurotransmitter metabolites, bile acids, C5-dicarboxylates, cAMP, cGMP, conjugated steroid hormones, prostaglandins, urate	Cimetidine, benzylpenicillin, bumetanide, cephalosporins, ciprofloxacin, furosemide, methotrexate, NSAIDs, pravastatin, probenecid, rosuvastatin, zidovudine
OAT	Oat	*SLC22A9*	OAT7	Liver	Conjugated steroid hormones, monocarboxylates, short chain fatty acids	Pravastatin
OAT	Oat	*SLC22A10*	OAT5	Liver	Unknown	
OAT	Oat	*SLC22A11*	OAT4	Kidney, placenta, adrenal gland	Bile acids, C5-dicarboxylates, conjugated steroid hormones prostaglandins, urate	Bumetanide, hydrochlorothiazide, ketoprofen, methotrexate, salicylate, tetracycline, torsemide, zidovudine
OAT	Oat	*SLC22A12*	URAT1	Kidney	Urate	
OAT	Oat	*SLC22A20*	OAT6	Nasal epithelial cells	Conjugated steroid hormones, C5-dicarboxylates, short chain fatty acids	Sorafenib
OAT	Oat	*SLC22A24*	S22AO	Kidney	Unknown	
OAT	Oat	*SLC22A25*	UST6	Liver	Unknown	
OAT	Oat-like	*SLC22A13*	OAT10	Kidney, brain, colon	C4-dicarboxylates, glutathione, nicotinate, urate	
OAT	Oat-like	*SLC22A14*	OCTL2	Testis, kidney	Unknown	

**Table 3:** Genetic variants in human organic cation transporters of the SLC22A family.

Transporter *(*Gene*)*	Genetic Polymorphism	Amino Acid Change	Effect on Expression	Activity	Effect on Drug Disposition
OCT1 (SLC22A1)	c.181C>T	R61C	=	Reduced	Reduced intestinal uptake of metformin
					Higher plasma concentrations of O-desmethyltramadol, sumatriptan, morphine, tropisetron, ondansetron
	c.252T>C	C88R	=	Reduced	Increased systemic exposure to sumatriptan
	c.1201G>A	G401S	=	Reduced	Reduced intestinal uptake of metformin
					Higher plasma concentrations of O-desmethyltramadol, sumatriptan, morphine, tropisetron, ondansetron
	c.1222A>G	M408V	=	Similar	Gastrointestinal side-effects of metformin
					Alteration of lamotrigine serum concentration
	c.1258_1260delATG	M420del	=	Reduced	Reduced intestinal uptake of metformin
					Higher plasma concentrations of O-desmethyltramadol, morphine, tropisetron, ondansetron
	c.1386A>C (rs622342)	-	↓Expected	Reduced	Low effect of metformin, levodopa
	c.1393G>C	G465R	=	Reduced	Reduced intestinal uptake of metformin
					Increased systemic exposure to sumatriptan
	OCT*5	G465R + M420del	=	Reduced	Higher plasma concentrations of O-desmethyltramadol, morphine, tropisetron and ondansetron
	OCT1*6	C88R + M420del	=	Reduced	Higher plasma concentrations of O-desmethyltramadol, morphine, tropisetron and ondansetron
	rs36056065 (c.1276 + 1ins GTAAGTTG)	-	Aberrant splicing		Gastrointestinal side-effects of metformin
OCT2 (SLC22A2)	c.495G>A	M165I		Reduced	
	c.596C>G	T199I		Reduced	Reduced renal clearance of metformin
	c.602C>T	T201M		Reduced	Reduced renal clearance of metformin
	c.808G>T	A270S		Reduced	Reduced renal clearance of metformin
	c.1198C>T	R400C		Reduced	
	c.1294A>C	K432Q		Similar	
OCT3 (SLC22A3)		A116S	=	Reduced	
		T400I	=	Reduced	
		A439V	=	Reduced	
OCTN1 (SLC22A4)	c.188G>A	R63H	↓Membrane	Reduced	
	c.248G>C	R83P	↓Membrane	Reduced	
	c.400C>A	L134M		Similar	
	c.475G>A	V159M		Similar	
	c.494A>G	D165G	↓Membrane	Lost	
	c.615G>A	M205I	↓Membrane	Reduced	
	c.774G>C	M258I		Similar	
	c.844C>T	K282X		Lost	
	c.917C>T	T306I	=	Similar	
	c.1031T>A	M344K		Similar	
	c.1445G>A	G482D	=	Reduced	
	c.1460T>C	M487T		Similar	
	c.1499T>A	I500N	=	Reduced	
	c.1507G>A	L503F	=	Reduced	Reduced tubular secretion of gabapentin
	c.1531G>A		=	Reduced	
OCTN2 (SLC22A5)	c.51C>G	F17L		Reduced	
	c.325G>C	E109Q		Similar	
	c.364G>T	D122Y	↓Membrane	Reduced	
	c.430C>T	L144F		Similar	
	c.523G>A	V175M		Similar	
	c.573G>T	K191N		Similar	
	c.614C>T	A214V		Similar	
	c.791C>T	T264M		Reduced	
	c.904A>G	K302E	↓Membrane	Reduced	
	c.934A>G	I312V		Similar	
	c.949G>A	E317K		Higher	
		M352R	=	Lost	
	c.1345T>G	Y449D		Reduced	
		P478L	=	Lost	
	c.1341TG>T	V481F		Reduced	
		V481I		Similar	
	c.1463G>A	R488H		Similar	
	c.1522T>C	F508L		Similar	
	c.1588A>G	M530V		Similar	
	c.1645C>T	P549S		Similar	

**Table 4:** Genetic variants in human organic anion transporters of the SLC22A family.

Transporter *(*Gene*)*	Genetic Polymorphism	Amino Acid Change	Effect on Expression	Activity	Effect on Drug Disposition
OAT1 (SLC22A6)	c.149G>A/C	R50H	=	Higher affinity for adefovir, cidofovir and tenofovir	No effect on renal clearance
	c.1361G>A	R454Q	=	Loss of activity	No effect on renal clearance
OAT2 (SLC22A7)	c.492_493insTCCCAG	E131_W132insSQ	=	Lost	Not investigated
	c.1592 + 206A>G	–	↑	Higher cell uptake	Anthracycline-induced cardiotoxicity and severe toxicity to capecitabine
OAT3 (SLC22A8)	c.445C>A	R149S	=	Lost	Not investigated
	c.715C>T	N239X	=	Lost	Not investigated
	c.779T>G	I260R	=	Lost	Not investigated
	c.829C>T	R277W	=	Reduced	Not investigated
	c.913A>T	I305F	=	Reduced	Lower renal clearance of cefotaxime
OAT4 (SLC22A11)	c.86T>C	L29P	=		Lower renal clearance of torsemide
	c.142C>T	R48Y	=	Reduced	
	c.464T>G	V155G	=	Reduced	
	c.1175C>T	T392I	=	Reduced	
OAT7 (SLC22A9)	c.268C>T	R90C	=	Reduced	Reduced hepatic uptake of pravastatin
	c.1298C>G	T433R	=	Reduced	
	c.1298C>T	T433M	=	Reduced	
	c.1437A>G	I479M	=	Reduced	
URAT1 (SLC22A12)	c.269G>A	R90H	=	Lost	Lower renal clearance of drugs substrates of the transporter
	c.412G>A	V138M	=	Lost	
	c.490G>A	G164S	=	Reduced	
	c.650C>T	T217M	=	Lost	
	c.774G>A	W258X	=	Lost	
	c.889C>T	Q297X	=	Lost	
	c.894G>T	E298D	=	Lost	
	c.1145A>T	Q382L	=	Reduced	
	c.1289T>C	M430T	=	Reduced	
	c.1639_1643del	V547Kfs	=	Lost	
	IVS2 + 1G>A	–	Aberrant splicing	Lost	
